# Dynamics of EEG Microstates Change Across the Spectrum of Disorders of Consciousness

**DOI:** 10.1007/s10548-025-01142-x

**Published:** 2025-09-13

**Authors:** Dragana Manasova, Yonatan Sanz Perl, Nicolas Marcelo Bruno, Melanie Valente, Benjamin Rohaut, Enzo Tagliazucchi, Lionel Naccache, Federico Raimondo, Jacobo D. Sitt

**Affiliations:** 1 Sorbonne Université, Institut du Cerveau - Paris Brain Institute - ICM, CNRS, Inserm, Paris, 75013 France; 2 Université de Paris Cité, Paris, France; 3 Department of Information and Communication Technologies, Centre for Brain and Cognition, Computational Neuroscience Group, Universitat Pompeu Fabra, Barcelona, Spain; 4 Department of Physics, University of Buenos Aires), Buenos Aires, Argentina; 5 National Scientific and Technical Research Council (CONICET), Buenos Aires, Argentina; 6 AP-HP, Hôpital de la Pitié Salpêtrière, Neuro ICU, DMU Neurosciences, Paris, France; 7 Latin American Brain Health Institute (BrainLat), Universidad Adolfo Ibáñez, Santiago, Chile; 8 Service de Neurophysiologie Clinique, AP-HP, Hôpital Pitié-Salpêtrière, Paris, France; 9 Institute of Neuroscience and Medicine (INM-7: Brain and Behaviour), Research Centre Jülich, Jülich, Germany; 10 Institute of Systems Neuroscience, Heinrich Heine University, Düsseldorf, Germany

**Keywords:** Disorders of consciousness, EEG microstates, Dynamics, Entropy production

## Abstract

As a response to the environment and internal signals, brain networks reorganize on a sub-second scale. To capture this reorganization in patients with disorders of consciousness (DoC) and understand their residual brain activity, we investigated the dynamics of electroencephalography (EEG) microstates. EEG microstates are meta-stable topographies that last tens to a few hundreds of milliseconds and are hypothesized to reflect large-scale cortical networks. To obtain EEG‑microstate segmentation, EEG topographies per sample were clustered into four groups for the purpose of the present comparison with the existing four‑class literature. We then obtained a time series of maps with different frequencies of occurrence and duration. One such occurrence of a map with a given duration is called a microstate. The goal of this work was to study the static and dynamic properties of these topographical patterns in DoC patients. Using the microstate time series, we calculated static and dynamic markers. In contrast to the static, the dynamic metrics depend on the specific temporal sequences of the maps. The static measure map coverage showed differences between healthy controls and patients. In contrast, some dynamic markers captured inter-patient group differences. The dynamic markers we investigated are Mean Microstate Durations (MMD), Microstate Duration Variances (MDV), Microstate Transition Matrices (MTM), and Entropy Production (EP). The MMD and MDV decreased with the state of consciousness, whereas the MTM non-diagonal transitions and EP increased. In other words, DoC patients had slower and closer to equilibrium (time-reversible) brain dynamics. In conclusion, static and dynamic EEG microstate metrics differed across consciousness levels, with the latter having captured the subtler differences between groups of patients with DoC.

**Supplementary Information:**

The online version contains supplementary material available at 10.1007/s10548-025-01142-x.

## Introduction

Consciousness is hypothesized to arise out of complex network interactions on a sub-second scale (Bodien et al. 2017; Seth and Bayne 2022). Various theoretical models build on the idea that conscious states do not rely on a single cortical area or network but require brain-wide communication (Seth and Bayne 2022). It has been argued that, in patients with Disorders of Consciousness (DoC), the network coordination is altered (Bodien et al. 2017), and thus the characterization of these pathological network dynamics can aid diagnostic and prognostic analyses. Addressing this challenge, Demertzi and colleagues (Demertzi et al. 2019), have demonstrated that different recurrent patterns of brain activity, obtained from resting-state fMRI, are directly associated with DoC clinical categories. Particularly, one of the phase-coherence patterns more present in higher global states of consciousness is characterized by long-range functional communication between brain areas (Demertzi et al. 2019).

The exploration of resting-state brain activity relies on the theory that the resting-state networks reflect an inner state of exploration which optimizes the system for input and thus it influences perception and cognitive processing (Deco et al. 2011). However, to respond to changes in the environment, networks must reorganize on a sub-second time scale. Unlike fMRI, the EEG temporal resolution can capture fast fluctuations. Using high-density EEG while tracing the ongoing brain activity of patients with a disorder of consciousness (DoC), we apply a method known as EEG microstates as a proxy to track latent brain state changes on a sub-second scale.

EEG microstates are defined as successive short periods (around 50–100 ms) during which the configuration of the scalp potential field remains semi-stable (Lehmann 1987; Michel and Koenig 2018). As reported in the literature (Brodbeck et al. 2012; Comsa et al. 2019; Kuhn et al. 2015; Michel and Koenig 2018; von Wegner et al. 2017; von Wegner and Laufs 2018), four prototypic maps can be reliably identified across healthy participants. These four maps are commonly denoted with letters and have distinct topographical descriptions: map A has a left-right orientation, map B has a right-left orientation, C has an anterior-posterior orientation, and D has a frontocentral maximum. The temporal characteristics of microstates are proposed to represent the basic building blocks of spontaneous mental processes, as well that the quality of mentation is determined by their occurrence and temporal dynamics (Michel and Koenig 2018). Furthermore, EEG microstates have been investigated in neurological and clinical conditions such as narcolepsy, Alzheimer’s, DoC, and schizophrenia (Kuhn et al. 2015; Michel and Koenig 2018; Stefan et al. 2018). The dominance of certain maps in time has also been associated with the activation of an underlying resting state network (Britz et al. 2010).

To assess the dynamics of the microstates in patients with a DoC compared to healthy controls (HC), we used the EEG recordings of the auditory local-global (LG) paradigm (Bekinschtein et al. 2009). The DoC clinical diagnostic categories with which we work in this paper are the Unresponsive Wakefulness Syndrome (UWS) (also known as the Vegetative State - VS), the Minimally Conscious State (MCS), and the Emergent Minimally Conscious State (EMCS) (Giacino et al. 2002, 2009; Jennett and Plum 1972). Recent work has also investigated the use of EEG microstates in the classification of DoC patients (Gui et al. 2020; Stefan et al. 2018; Toplutaş et al. 2024). For diagnosis, the percentage of time spent in microstate D in the alpha range was found to be informative (Stefan et al. 2018). The map percentage of occurrence differed between patients and HC (Gui et al. 2020) as well as their mean duration (Toplutaş et al. 2024). The duration of microstate A in the delta band, the frequency of map A in the theta and the 2–20 Hz band were found to be indicators of prognostic improvement (Stefan et al. 2018). However, neither study investigated the dynamic microstate markers in detail.

The first working hypothesis of this study was focused on the investigation of the microstate dynamics across DoC classes and aimed to test whether similar to the loss of consciousness in sleep there is a lengthening of the microstates across DoC patients. In other words, UWS patients will have significantly longer microstate durations than MCS, EMCS, and HC. Furthermore, we hypothesized that the variance of microstate durations would be highest for HC and lowest for UWS. Another approach to assess the change of dynamics is the concept of time-reversibility (or equilibrium dynamics) (Sanz Perl et al. 2021), in which we assessed whether the discrete state transitions are symmetrical when going from brain state A to brain state B and reversed - this is represented through a measure called entropy production. Recent work measured the entropy production in fMRI acquisitions in humans doing multiple cognitive tasks (Lynn et al. 2021) and in electrocorticography and fMRI recordings of various global states of consciousness in primates and humans (Sanz Perl et al. 2021). The authors showed that there is a departure from equilibrium dynamics that depends on the physical and cognitive demand of the task (Lynn et al. 2021), as well as that reduced consciousness is at a proximity of the dynamic equilibrium (Sanz Perl et al. 2021). The microstates, which reduce the dimensionality of the whole-brain activity and discretize it into four maps, allows us to determine whether some transitions from one map to another are more present in some of the investigated groups. We hypothesized that the transition probabilities matrices of the HC would show a lack of symmetry compared to the patient groups. In other words, the probability of transitioning from, for example, map A to map B would be different from the reverse.

## Methods

### Participants

The study involved two groups of participants: a group of patients with DoC, a healthy group used as a control for the analyses (*n* = 37, mean age 27.6 +/- 5.8, 8 female). According to the behavioral assessment by a neurologist using the CRS-R, the patients were classified to be in Unconscious Wakefulness Syndrome (UWS) otherwise known as Vegetative State (VS) (*n* = 70, mean age 46.4 +/- 18.1, 22 female), Minimally Conscious State (MCS) (*n* = 70, mean age 43.7 +/- 18.6, 28 female), Emergent MCS (EMCS) (*n* = 14, mean age 38.9 +/- 23, 4 female). The healthy participants took part in the study voluntarily as approved by the ethical committees at the Paris Brain Institute (ICM), Pitié Salpêtrière Hospital, Sorbonne University (Inserm CPP C13-632 41). The patients’ recordings were acquired as part of an operational diagnostic procedure at the Neurology Department at the Pitié Salpêtrière Hospital or as part of other studies aiming for the development of diagnostic and prognostic analyses (Engemann et al. 2018; Sitt et al. 2014). The research was approved by the ethical committee of the hospital under the French label of ‘routine care research’ (Comité de Protection des Personnes n◦ 2013-A01385-40, Ile de France 1, Paris, France under the code ‘Recherche en soins courants’, protocol number M-Neuro-DOC, CE SRLF 20 − 2).

### Experimental Design and Data Characteristics

The EEG data were recorded with a 256-electrode geodesic sensor net (EGI^®^, Oregon, USA) referenced to the vertex. The sampling rate was set to 250 Hz. For the goal of this study, we used recordings during an auditory LG paradigm (Bekinschtein et al. 2009). Specifically, we used the period of the paradigm during which four identical sounds are presented. We refer to this period as a pseudo-resting state as it elicits a non-specific response (Engemann et al. 2018). Furthermore, previous publications from our lab have demonstrated that the features extracted (at the single-trial level) don’t differ between pure resting-state data and the truncated segment of 4 equivalent sounds data (Engemann et al. 2018). In this pseudo-resting state period, the same auditory responses are elicited by patients and healthy controls (Bekinschtein et al. 2009). It is essential to highlight that the cognitive process of counting the deviant trials occurs after the onset of the fifth sound, which we don’t include in our analysis.

### Data Pre-processing

The MNE-Python (Gramfort et al. 2014) and the NICE-tools and extensions (Engemann et al. 2018), which are Python 3.7-based open-source software libraries, were used for the EEG signal processing. The recordings were band-pass filtered (0.5–45 Hz) then segmented in epochs ranging from − 200 ms to + 1344 ms relative to the first sound onset. The data was epoched to enable better artifact cleaning where single strongly artefacted epochs were removed. Similar approaches have been taken in other studies (Milz et al. 2016; Sikka et al. 2020). Electrodes with voltages exceeding 100 µV in more than 50% of the epochs were removed. Moreover, voltage variance was computed across all correct electrodes. Electrodes with a voltage variance Z-score higher than 4 were also removed. This process was repeated four times. Bad electrodes were interpolated using a spline method. Epochs were labeled as bad and discarded when the voltage exceeded 100 µV in more than 10% of electrodes. The remaining stimulus-locked epochs were re-referenced to an average reference. In addition, the channels along the edges of the head were removed systematically from all the recordings. This was done due to the electrodes containing face muscle artifacts or not having sufficiently strong signals. In the cases where more of the 50% of the epochs were marked as bad, the participant was then taken out of the analysis. They are not included in the patient summaries above.

### Microstates Analysis

EEG microstates have been studied for more than 30 years (Lehmann 1987; Michel and Koenig 2018). Nevertheless, there are still ongoing debates on the subtleties regarding their derivation and analysis. In this study, a combination of the procedures most widely accepted in the scientific community was followed (Brodbeck et al. 2012; Comsa et al. 2019; Michel et al. 2009; Michel and Koenig 2018; Poulsen et al. 2018; Stefan et al. 2018; von Wegner et al. 2017; von Wegner and Laufs 2018; Zanesco et al. 2020). We used a modified version of the MNE microstates package^1^ as well as metrics introduced initially in other studies (von Wegner et al. 2017; von Wegner and Laufs 2018).

### Microstates Clustering and Segmentation Methods

**Fig. 1:**
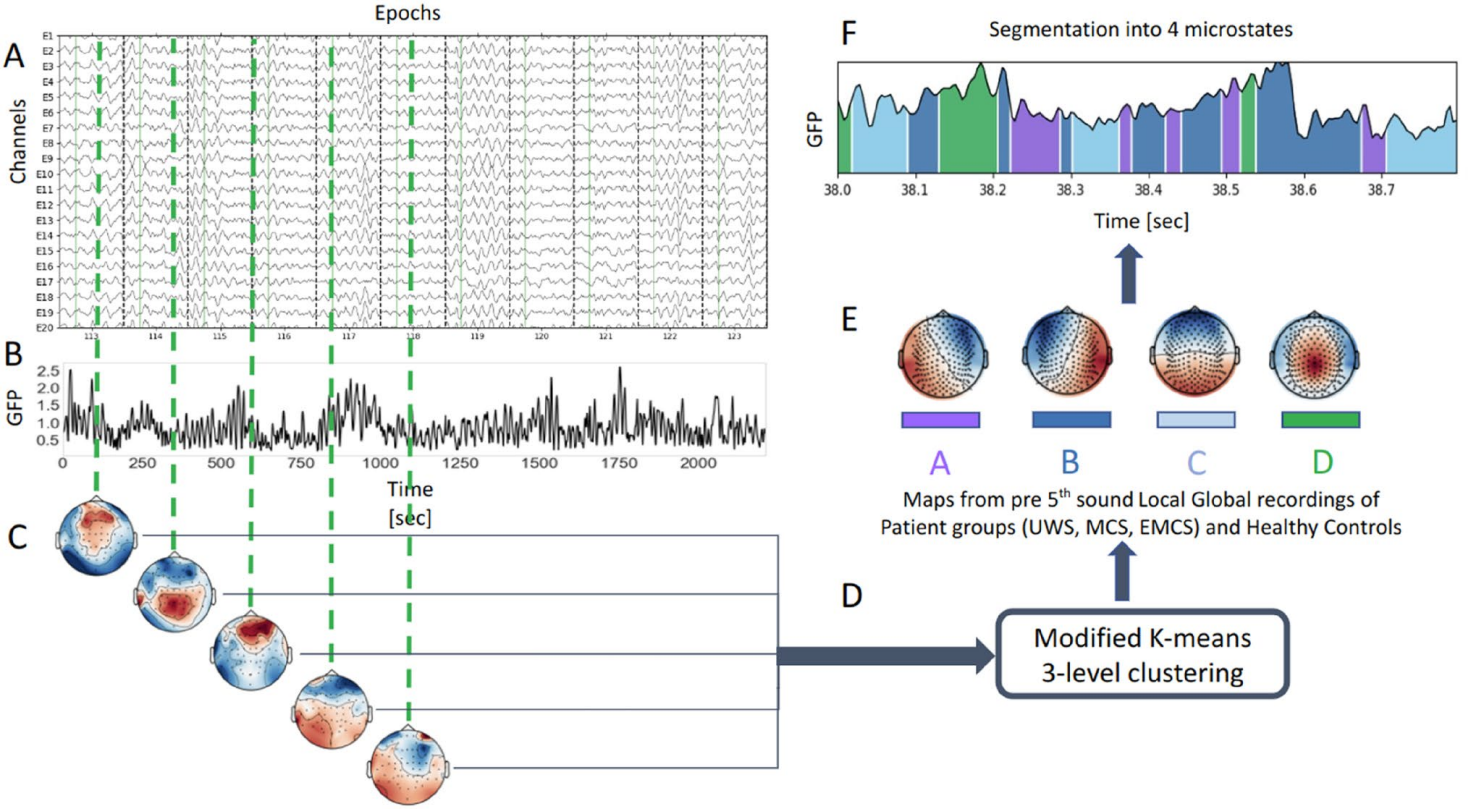
Illustration of the microstates clustering and segmentation algorithms. **(A)** The participants’ EEG is divided into epochs (a sample of epochs and channels is shown). **(B)** The Global Field Potential (GFP) is calculated by taking the EEG signals from all the electrodes at each time point. **(C)** The GFP peaks are extracted and filtered according to two criteria (explained in the Materials and Methods). The topographies (EEG potentials from all the electrodes) are extracted at each GFP peak. **(D)** The extracted topographies at the GFP peaks are used as input to a modified k-means clustering algorithm. **(E)** The output of the clustering gives a k-number of topographies that explain most of the variance in the data. Each topography or microstate map is denoted with a different color - shown in the square below the maps. **(F)** The maps are fitted back to the EEG channel signal to obtain the sequence of dominant maps along with the duration of the recording, this is known as segmentation. Abbreviations: Global Field Power (GFP), seconds (sec), Vegetative State (VS), Unresponsive Wakefulness Syndrome (UWS), Minimally Conscious State (MCS), Emergent Minimally Conscious State (EMCS)

The Global Field Power (GFP) of the signal was calculated which quantifies the variance of voltage potentials across all of the electrodes (Pascual-Marqui et al. 1995). As a measure of the strength of the scalp potential at a given time point, the GFP is based on the potential differences between all electrodes. The output is a vector of scalar values per sample. (Here by sample we referred to one momentary record of the EEG data, in this case we had 250 samples per second or the acquisition of EEG data at 250 Hz, whereas epochs are time-windows extracted from the EEG signal.) In the past, high GFP has been associated with stable EEG topographies (Brodbeck et al. 2012; Michel et al. 2009). The maps were traditionally seen as discrete, meaning they do not gradually morph into one another or overlap in time, but rather a single map is dominant and then abruptly transitions to another map (Lehmann 1987). However, this has been disputed in recent studies (Mishra et al. 2020; Shaw et al. 2019). To obtain the time series of microstates, the EEG epochs from a single participant were stacked going from a 3D array (epochs, channels, samples) to a 2D array (channels, samples). For our goal, we extracted the EEG potential fields at the GFP maxima (the minimal peak distance was set to 2 samples), also referred to as maps (Fig. 1). The topographies were filtered according to two criteria. The first filtering was done to remove the maps at GFP peaks that were larger than one standard deviation above the GFP mean (as suggested in (Poulsen et al. 2018). Secondly, the maps that belong to a GFP local maxima but within the lowest 15% of the GFP signal were removed. As shown by Mishra et al. (2020), in this GFP range, the k-means clustered maps were equidistant from the EEG potential fields at the given sample. The remaining GFP peaks were subsampled randomly to less than 100.000 maps per recording. This was done to optimize the computations. The remaining maps were submitted to a modified k-means clustering algorithm as described by Pascual-Marqui et al. (1995). The modified k-means clustering algorithm and the segmentation functions were available as a sub-package of MNE Python (Gramfort et al. 2014). The specificity of this algorithm is that it is polarity-invariant, meaning that topographies with opposite polarity are assigned to the same class (Pascual-Marqui et al. 1995). Not all clustering algorithms are polarity invariant, one such example is classical k-means clustering. In this work, the number of clusters (k) was set to four to compare the results to existing literature, as in most studies this is the chosen number of clusters (Brodbeck et al. 2012; Comsa et al. 2019; Kuhn et al. 2015; Michel and Koenig 2018; Stefan et al. 2018).

To assess to what extent the maps explain the data, a measure called Global Explained Variance (GEV) was used (Eq. 1). The first step was to fit the map closest to the EEG field potential at the given time point. This was done for each sample of the EEG signal and the output 1D vector (map per sample) was denoted as a segmentation. In other words, the maps were back-fitted to new EEG samples based on topographical similarity. The segmentation gives the information on which map is closest to the topography sample by sample. The topographical similarity was calculated based on a distance measure called Global Map Dissimilarity (GMD) (Murray et al. 2008). This measure looks at how similar the topography maps are and is invariant to the strength of the signal. In other words, two maps with similar topographies, but different EEG voltage strengths will result in a low GMD distance (Murray et al. 2008).

GEV is a measure of the similarity of each EEG sample to the microstate map it has been assigned to. It was calculated by the multiplication of the squared correlation between the EEG sample and the assigned map with the sample’s fraction of the total squared GFP (Murray et al. 2008). The k-means clustering was randomly initiated 100 times, and at each iteration, the GEV and the corresponding maps were saved. This was done to maximize the GEV by selecting the maps from the iteration with the highest GEV value.

For a group-level analysis, we did a three-level clustering (Supplementary Fig. 3). First, we clustered into 10 maps on a participant level. Next, because of the unbalanced dataset (e.g. 70 MCS participants versus 14 EMCS), we performed bootstrapping. In other words, we sampled from the groups with repetition. The sample size was equal to the smallest group size, in our case it was 14 as we have only 14 patients in an EMCS. In each bootstrap iteration, we clustered the participant-level maps into four maps. We repeated this 2000 times. We obtained an array of 4 × 2000 maps, which were clustered into 4 maps. The final four maps were the ones we used to fit back to the participant time series to obtain the per-participant segmentation. When a given map is dominant over a few continuous (uninterrupted) samples, this is what we call a microstate. Using this time series (the segmentation) we calculated the microstates markers. A similar but simpler double clustering procedure has been previously used (Sikka et al. 2020; Zanesco et al. 2020).

Another method used to deal with EEG noise is to smoothen the segmentation. Due to various short-lived artifacts of a few samples, the back-fitting of the maps to the EEG can be affected. For this reason, the segmentation was smoothened using a window smoothing algorithm explained in Titterington et al. (1985) (introduced in Pascual-Marqui et al. (1995) and implemented in the MatLab EEG microstates toolbox by Poulsen et al. (2018).

### Microstates Markers

For the goal of this study, we investigated markers calculated from the microstate segmentation (Brodbeck et al. 2012; Michel and Koenig 2018; von Wegner et al. 2017). We divided the microstate markers into static and dynamic. The static metrics are time-independent, meaning they are not influenced by the specific temporal sequence of the microstates. They are:

- Map coverage, sometimes referred to as segment count density, or empirical symbol distribution (von Wegner et al. 2017; von Wegner and Laufs 2018). It denotes the fraction of the total time for which a certain microstate is dominant. In other words, it indicates the percentage of time covered by a given microstate map over the duration of all the epochs.
- GEV per map reflects the ratio of variance explained by each of the k-group maps - calculated for all the epochs of one participant. This reflects the goodness of fit of the microstates to describe the map sequence of that participant. The markers GEV and map coverage depend on one another. Meaning that the bigger the coverage is, the more variance the given map is likely to explain within the data. Contrastly, the GEVs of the maps reflect the topographical similarity to each data point (how well they correlate), whereas the map coverage accounts for the discrete temporal presence without reflecting the correlation between the dominant map and the given sample (Kuhn et al. 2015).

The dynamic markers which depend on the temporal sequence of map alterations are:

- Mean Microstate Duration (MMD) represents the mean of the microstate durations in milliseconds per participant. This marker gives information on the stability of the microstates and can allow for the comparison of whether microstate transitions occur faster or slower in a given group of participants.
- Microstate Duration Variance (MDV) represents the variance of the microstate durations per participant. It reflects the microstates’ duration variability, and their temporal consistency or lack thereof. In other words, it shows whether the durations of the microstates within a participant are consistently long, or consistently short, or if they have a variable length.
- Microstates Transition Matrix (MTM) describes the transition between the k-microstates. It denotes the ratio of the number of transitions from one microstate map to another and is calculated from the transitions present in all the epochs from a single participant’s EEG recording. The transition probabilities quantify how frequently a given map is followed by the other maps. It characterizes the flow of microstates in terms of particular sequences of transitions and allows one to determine if this sequence has a particular dynamical order.
- Entropy Production (EP) quantifies the symmetry of the transition probabilities. It is a measure of broken detailed balance (where the transition probability of going from map i to map j (Pij) is different than the transition probability going from map j to map i (Pji) and this difference leads to EP increase). This analysis has been implemented in previous fMRI studies in resting state and task paradigms (Lynn et al. 2021; Sanz Perl et al. 2021).

1 $$\:EP={\sum\:}_{ij}^{}{P}_{ij}log\frac{{P}_{ij}}{{P}_{ji}}$$

### Statistical Analyses

Due to the microstate marker distributions per group being skewed, we performed non-parametric statistical tests. To investigate the differences per pair of groups, the Mann-Whitney U test was used for comparing independent data samples. This test is a nonparametric version of the independent samples t-test. Its null hypothesis stipulates that the two groups come from the same population. To control for the type 1 error rate, the Bonferroni corrected p-values were analyzed. The significance level, alpha, was set to 0.05 for all statistical tests.

## Results

Our primary focus was on the validation of the method by comparing the EEG microstate topographies of patients with DoC to those of HC from this study and previous ones. Secondly, we investigated the static microstate markers, specifically the map coverage and GEV. The third part focused on dynamic microstate metrics, specifically the MMD and MDV, transition matrices (TM), and entropy production. The findings highlight microstate differences between HC, patients, and across patient groups.

### EEG Microstates in Patients with Disorders of Consciousness

**Fig. 2:**
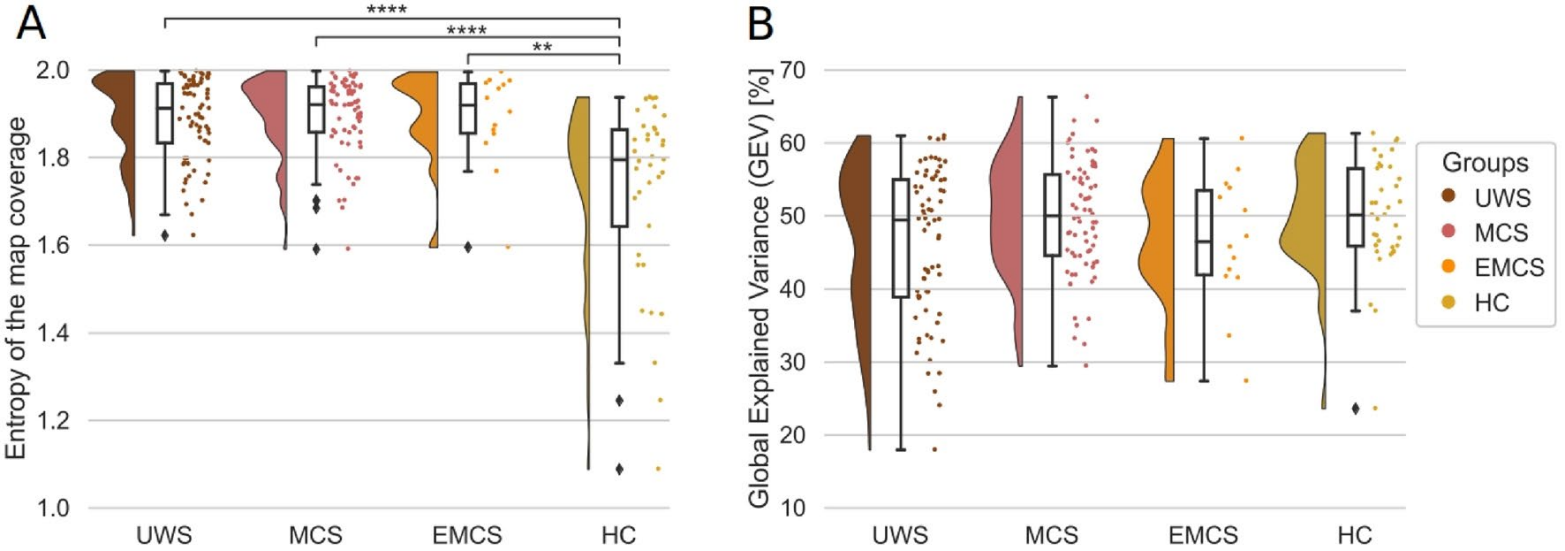
The static microstate metrics Entropy of the map coverage covered differs between patients with Disorders of Consciousness and Healthy Controls. **(A)** Entropy of the map coverage covered by the microstates for all three patient groups and the HC. **(B)** Summed GEV for all microstates together, plotted separately for all three patient groups and the HC. According to the Mann-Whitney U tests, the map coverage values between some of the groups are statistically different. All values are Bonferroni corrected. In the figures, one dot represents one participant. The stars represent significance following Mann Whitney U tests between distributions (* *p* < 0.05; ** *p* < 0.01; *** *p* < 0.001; **** *p* < 0.0001). Abbreviations: map coverage, Global Explained Variance (GEV), Unresponsive Wakefulness Syndrome (UWS), Minimally Conscious State (MCS), Emergent Minimally Conscious State (EMCS), Healthy Controls (HC)

The EEG microstates depict generalized topographical alterations (Fig. 1) whose occurrence and stability are associated with the same or close-by neural sources. The method involves the clustering and segmentation of EEG microstates, where the EEG data is divided into epochs, the GFP peaks are extracted and filtered, and a modified k-means clustering algorithm is applied to identify topographies representing microstate maps, which are then fitted back to the EEG signal to obtain the sequence of dominant maps and their durations. The first question when studying these topographies in patients with DoC is whether they will be different from the ones of the HC observed both in this study and previous ones. The final maps, derived from the three-level clustering (Supplementary Fig. 3) shown in Fig. 1. E, had visually similar topographic organization as the ones reported in the literature (Brodbeck et al. 2012; Comsa et al. 2019; Kuhn et al. 2015; Michel and Koenig 2018; Toplutaş et al. 2024; von Wegner et al. 2017; von Wegner and Laufs 2018). Additionally, the GEV did not differ between the patient groups and HC (Fig. 2B).

### Static Microstate Markers

Two measures to assess the static properties of the microstates were the map coverage and GEV (see Methods). To summarize the occupancy ratios of each map, we calculated their entropy. When looking into the entropy of the map coverage or the ratio of the dominance of one map, in Fig. 2 we observed differences between the HC and all the patient groups, but no differences among the patients’ groups (between UWS and HC U(70,37) = 2031, *p* < 0.0001; between MCS and HC U(70, 37) = 2104, *p* < 0.0001; between EMCS and HC U(14, 37) = 418, *p* = 0.0049). The healthy participants, on a group level, showed a decrease in entropy, meaning that the occurrences of the four maps were more predictable. Whereas the patient groups had higher entropies showing lower predictability of transitions (or in other words close to uniform probabilities of 25% for the 4 maps). This indicated that some maps, especially in the HC, dominated. To understand this trend we analyzed the coverage per map and per group (Supplementary Fig. 1). We observed differences in the distributions between HC and the patient groups for maps A, B, and C (Supplementary Fig. 1, and Supplementary Table 1). Maps B, C, and D were less present in HC, and map C was more present (Supplementary Fig. 1, and Supplementary Table 2). We only saw a similar trend between GEV and coverage for map C (Supplementary Fig. 1). These differences in coverage in all maps for the HC were what contributed to the lower entropy values.

In Fig. 2B the summed GEV of all four maps per participant group is shown. Following Mann-Whitney U two-sided tests, no significant differences between the pairs of groups were observed (*p* > 0.05, Bonferroni corrected). Thus, the ability of the four clustered maps to capture the variance of the true sample-to-sample topographies did not significantly differ between HC and DoC patients. In other words, maps did not bias the dynamic markers towards one of the included groups. When we analyzed the GEV separately for each map (Supplementary Fig. 1B), we only observed differences between some of the patient groups and the HC in maps B and C, and between UWS and MCS in map A (Supplementary Fig. 1, and Supplementary Table 1). The inter-group differences reflected the same trends in GEV and map coverage which was expected given they revolve around the closeness of the attributed map and the original topography (see Methods). In the literature, similar GEV values are reported (Brodbeck et al. 2012; Comsa et al. 2019; Zanesco et al. 2020), roughly around 10–15% per microstate map, with higher values, above 20%, for the anterior-posterior microstate C, which was something that we also observed.

### Dynamic Microstate Markers

**Fig. 3:**
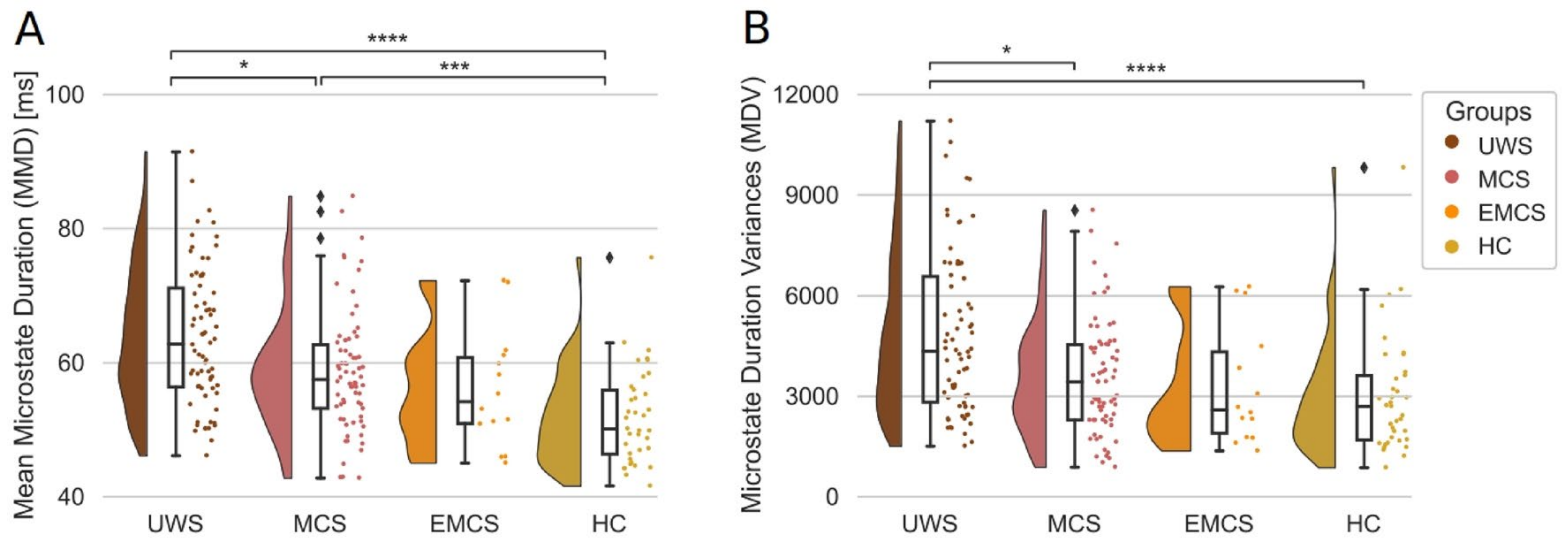
Dynamic microstate markers: MMD and MDV differ between Disorders of Consciousness groups and Healthy Controls. **(A)** MMD for all microstates for all three patient groups and the HC. Statistical differences are found between the HC and the three patient groups. **(B)** Same as (A) but for the MDV. The statistic and the p-value of the Mann-Whitney U two-sided tests for the MMD and the MDV are given in the Results. In all the panels, one dot represents one participant. All p-values are Bonferroni corrected. The stars represent significance following Mann Whitney U tests between distributions (* *p* < 0.05; ** *p* < 0.01; *** *p* < 0.001; **** *p* < 0.0001). Abbreviations: Mean Microstate Duration (MMD), Microstate Duration Variance (MDV), milliseconds (ms), Unresponsive Wakefulness Syndrome (UWS), Minimally Conscious State (MCS), Emergent Minimally Conscious State (EMCS), Healthy Controls (HC)

The dynamic microstate markers showed higher inter-group differences (Fig. 3). Both the MMD and the MDV, on a group level, showed a decreasing trend. In other words, going from the patient group UWS to HC, we saw a decrease in the microstate durations. Similarly, there was a decrease in the variance of durations which reflects an increased consistency across microstate durations going from UWS to HC, with a plateau between the EMCS and HC. We test this decrease using Mann-Whitney U tests, two-sided and Bonferroni corrected. The differences were significant between the groups for the MMD: UWS and HC U(70,37) = 2174, *p* < 0.0001, MCS and HC U(70, 37) = 1892, *p* = 0.0006, UWS and MCS U(70,70) = 3158, *p* = 0.019. Following the Bonferroni correction, the non-significant pairs were: EMCS and HC U(14, 37) = 335, *p* = 0.066; UWS and EMCS U(70,14) = 700, *p* = 0.07; MCS and EMCS U(70,14) = 578, *p* = 1. For the MDV we observed the intergroup differences: UWS and HC U(70,37) = 1950, *p* = 0.0001; and UWS and MCS U(70,70) = 3176, *p* = 0.015. Following the Bonferroni correction, the non-significant pairs were: MCS and EMCS U(70,14) = 546, *p* = 1; MCS and HC U(70, 37) = 1606, *p* = 0.25; EMCS and HC U(14, 37) = 292, *p* = 1; UWS and EMCS U(70,14) = 690, *p* = 0.099. Visually we can observe a gradient of durations and variances as a function of consciousness state, thus we tested its significance using a Spearman correlation. For the MMD, the relationship with the groups was significant with a rs(189)=−0.54, *p* < 0.0001, and similarly for the MDV it was rs(189)=−0.45, *p* < 0.0001.

When we looked into the MMD and MDV separately per each map (Supplementary Fig. 2, and Supplementary Table 3), we saw that the trend was consistent. For map D, we observed a lower microstate duration especially in the HC as was expected from the lower coverage values of map D for this group. The higher intra-participant variance in the UWS patients reflected an instability in the duration of the microstates to a higher degree. Conversely, as HC showed the lowest intra-participant variance, we can state that the microstates’ duration was more consistent in this group and consistently short as shown by the mean microstate durations.

**Fig. 4:**
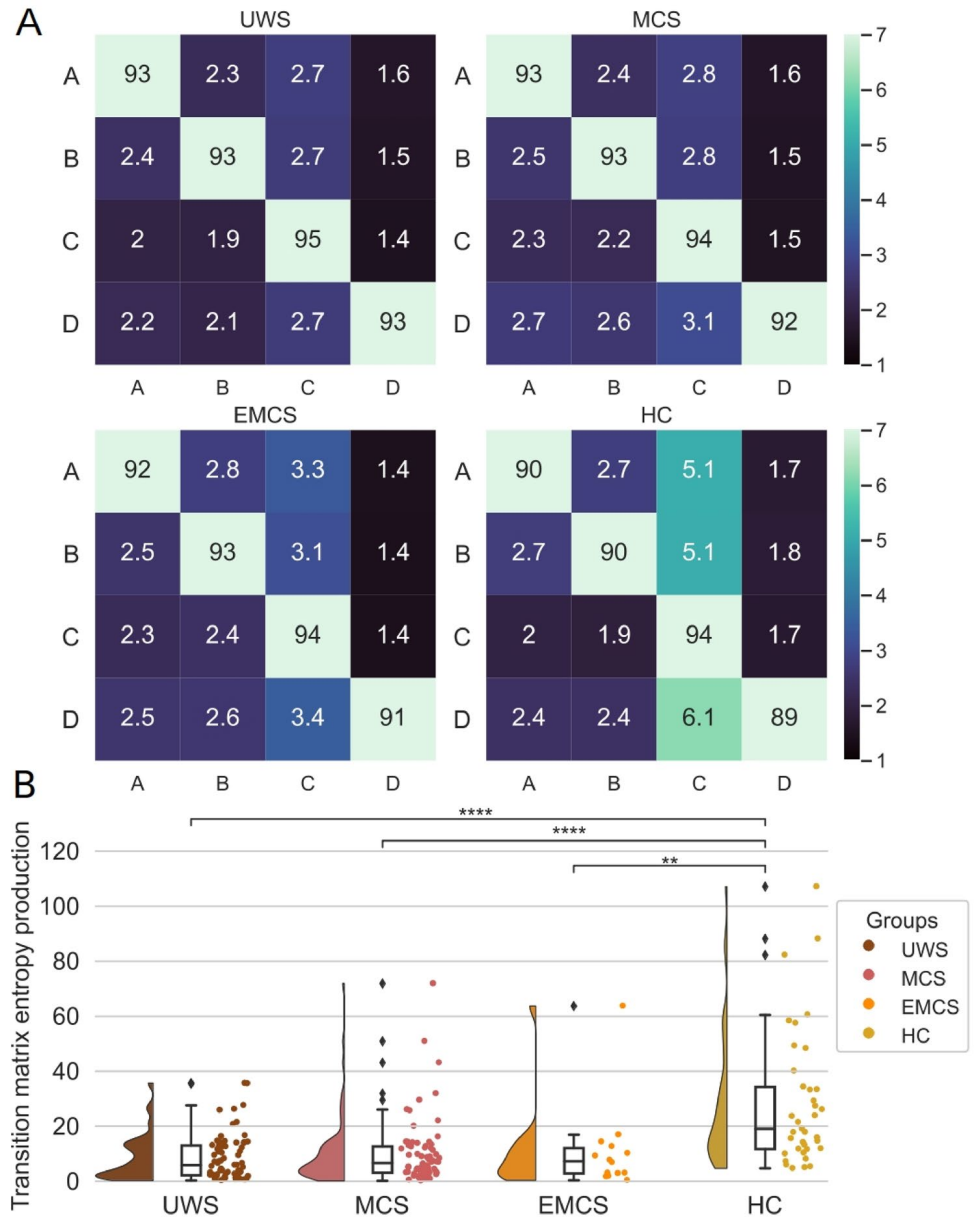
The MTM summarized using the Entropy productions show differences between Healthy Controls and patients with Disorders of Consciousness. **(A)** The MTM transitions are shown going from a row value to a column value. The transitions are given in percent. (**B)** Entropy productions per group of the transition probabilities. According to the Mann-Whitney U tests, significant differences were found between the HC and each of the three patient groups. The stars represent significance following Mann Whitney U tests between distributions (* *p* < 0.05; ** *p* < 0.01; *** *p* < 0.001; **** *p* < 0.0001). All values are Bonferroni corrected. In the last panel, one dot represents one participant. Abbreviations: Unresponsive Wakefulness Syndrome (UWS), Minimally Conscious State (MCS), Emergent Minimally Conscious State (EMCS), Healthy Controls (HC), Microstates Transition Matrices (MTM)

In Fig. 4 the average transition probabilities going from map X (row) to map Y (column) are given per group. For the patient groups, we saw more than 90% of self-transitions. This is aligned with the longer durations we observed in the patient groups. In addition, we observed a decrease in symmetry $\:({P}_{ij}\ne\:{P}_{ji})$. This indicates that there were some memory effects in the transitions or non-reversible dynamics (Lynn et al. 2021; von Wegner et al. 2017; von Wegner and Laufs 2018). Another way to represent the information captured by the transition matrices is entropy production (Lynn et al. 2021; Sanz Perl et al. 2021). The transition matrix entropy production measure per group is given in Fig. 4B where the Mann-Whitney U two-sided tests showed significant differences between the HC and the patient groups (UWS and HC U(70,37)=433, *p* < 0.0001; MCS and HC U(70, 37) = 471, *p* < 0.0001; EMCS and HC U(14, 37)=96, *p* = 0.0036). This showed that the entropy production is higher in healthy participants than in patients. Indicating that the HC microstate transitions were further from an equilibrium compared to the patient groups.

## Discussion

### Summary

In this study, we used a pseudo-resting-state EEG analysis aimed to investigate the temporal characteristics of ongoing spatial patterns. The configuration of scalp topographies remained semi-stable over successive short-time periods lasting on average from 50 to 100 milliseconds. These periods of stability are what we call microstates. Four canonical maps and their temporal properties have been hypothesized to represent the quality of mentation in resting state recordings (Michel and Koenig 2018). However, so far, these topographies have been most often analyzed from low-density EEG recordings. In this study, a high-density (256 electrodes) EEG system was used, which can capture more detailed topographies. In this case, the maps must be precise enough to separate functionally different states (for example in UWS and HC), but also generalizable enough to allow cross-group comparisons (Michel and Koenig 2018). The k-means clustering output resulted in maps that can be reliably compared with the canonical maps typically reported in the literature. A group-level clustering was conducted on all DoC and HC groups together and two groups of markers were investigated, static (map coverage and GEV) and dynamic (MMD, MDV, TM, and EP). The markers map coverage, the GEV, and the MMD derived from the HC microstates segmentation, are comparable with other studies (Comsa et al. 2019; Gramfort et al. 2014; Koenig et al. 2002; Michel and Koenig 2018; Toplutaş et al. 2024). We extended the analysis by using metrics that capture different properties of the distributions such as the MDV and EP.

### Static Microstate Markers Do not Differ Across Patient Groups

The static markers, which are not dependent on the temporal dynamics of the microstates, showed no reliable differences among patient group categories (Fig. 2). The low GEV of microstate D could reflect a higher heterogeneity of the topographies that were assigned to map D. Such a trend was observed by Brodbeck et al. (2012) but only in non-REM and deep sleep. Interestingly, map D had similar coverage values in UWS and MCS to the ones of other maps, and lower in one part of the HC group. Comsa et al. (2019) findings are aligned with our results, there seems to be a lower entropy, and less balanced coverage when participants are awake than when they are asleep.

On the contrary, map coverage entropy values in our study differed from those observed in another work with patients, where the coverage distributions in HC were more balanced, and less predictable, with a higher entropy; than the UWS and MCS patients (Gui et al. 2020). In another work on sleep, similarly, there was a decrease in entropy going from awake to deep sleep, with N2 sleep showing the highest coverage variance between maps (lowest entropy) (Brodbeck et al. 2012). However, any comparison between sleep and DoC as states of consciousness has to be taken with precaution because there is mounting evidence that sleep cannot be studied as a uniquely unconscious state, but rather shows high heterogeneity (Andrillon et al. 2016).

### Dynamic Microstate Markers Differ Across Patient Groups

When analyzing the MMD, we observed lower group-level values, going from UWS, MCS, to EMCS, and HC where there is a plateau. Furthermore, the shortening of EEG microstates in the HC, revealed faster dynamics in fully preserved consciousness in the HC. This is captured by other studies that investigated EEG microstates in a smaller sample of DoC patients with an average of around 20 ms lengthening of the microstates duration (Toplutaş et al. 2024), in drowsy and asleep participants, with 10 ms MMD lengthening is asleep compared to awake and attentive participants (Comsa et al. 2019), and 50 ms lengthening in deep sleep compared to wakefulness (Brodbeck et al. 2012). In other words, the microstate durations during deep sleep were twice as long compared to when the participants were awake; and the deeper the participants were asleep, the slower the topographical alterations were. Furthermore, the average duration of microstate D was significantly increased when participants in the study were drowsy or had fallen asleep in comparison to when they were awake (Comsa et al. 2019). A parallel that can be drawn between the studies done on sleep and DoC patients. Sleep, which is a transient and heterogeneous loss of consciousness, and DoC diagnostic classes which reflect a non-reversible loss of consciousness condition, both showed similar microstate dynamics.

Similarly, in the MDV distributions, a lowering of per-participant variance scores from UWS to HC was observed. The group pairwise Mann-Whitney U tests, after a Bonferroni correction, revealed significant differences between some pairs of groups (Fig. 3). The initial hypothesis we postulated is that the HC group will show the highest per-participant variance. The line of thought is that the consciousness level, and thus inter-participant differences would be reflected in a higher variance of microstate durations. A variance that should not show up in patient groups. Similar variances (spread) of the MMD distributions were reported when participants are asleep compared to when they are awake (Brodbeck et al. 2012; Comsa et al. 2019), especially when contrasting deep (N3) sleep with wakefulness (Brodbeck et al. 2012). However, in both studies, the participant-level MDV was not calculated, and a direct comparison entails further investigation.

When looking into the lower per-participant variance in HC, one could postulate that the microstates do not reflect inter-participant conscious content, but rather more general brain dynamics of conscious states. Furthermore, the higher variance in the patient groups could reflect the high heterogeneity of the etiologies and clinical pictures across patients. Another possibility is that in the patient groups, the microstate durations were consistently long, but because we use short epochs, their durations are interrupted.

### Entropy Production Is Lower in Patients Compared To Healthy Controls

The decrease in symmetrical transitions across states was captured by the measure of entropy production (Lynn et al. 2021; Sanz Perl et al. 2021). On a group level, the differences were significant between the HC and the three patient groups. This indicates that the microstates time series in the HC had higher irreversibility, or there is a breaking in detailed balance. In contrast, the patient groups showed entropy production values aligned with higher time reversibility. In each of the groups, there were a few patients who showed higher state transition asymmetry. Further investigations can test if the higher entropy production in patients is aligned with a better prognosis (Lynn et al. 2021; Sanz Perl et al. 2021). Our results were aligned with previous observations which report higher entropy production with an increase in task cognitive demand (Lynn et al. 2021) and a decrease in global states of unconsciousness (Sanz Perl et al. 2021). Other analogous metrics have been proposed, where a breaking of temporal symmetry (analogous to the entropy production) is shown in healthy control compared to patients with a disorder of consciousness (Guzmán et al. 2023).

## Conclusions

In this study, we explored the dynamics of EEG microstates in patients with DoC to understand residual brain activity and the reorganization of brain networks on a sub-second scale. By analyzing static and dynamic EEG microstate markers, we aimed to differentiate between HC and DoC patients and among different DoC groups. Our findings indicate that while static markers like the map coverage and GEV did not distinguish between patient groups, dynamic markers revealed significant inter-group differences. Specifically, the MMD and MDV decrease with higher consciousness levels, whereas non-diagonal transitions in MTM and EP increase. These results suggest that DoC patients exhibited slower and more equilibrium-like brain dynamics, reflecting a state closer to time-reversibility. This study enhances our understanding of brain dynamics in DoC patients by showing that dynamic EEG microstate metrics were more sensitive in capturing subtle differences in brain activity among patients with varying levels of consciousness, and translates entropy production measures to EEG acquisitions. We would like to emphasize that although the goal of our study was to compare the four canonical maps reported in microstates and states of consciousness research, future work should explore what is the optimal number of maps, either using a similar methodological approach as ours with a two-level clustering, or finding different maps per explored group. Further lines of research can also investigate the dynamic EEG microstate markers in movie watching or active tasks, where the differences across patient groups could be further enhanced, as expected from fMRI studies in healthy controls (Lynn et al. 2021).

## Supplementary Information

Below is the link to the electronic supplementary material.

Supplementary Material 1

## Data Availability

The data is not publicly available. For the analyses, we used open-source software MNE Python and the associated implementation of the EEG microstates analysis (https://github.com/wmvanvliet/mne_microstates*).*

## Abbreviations

DoC: Disorders of Consciousness
EEG: Electroencephalography
MMD: Mean Microstate Durations
MDV: Microstate Duration Variances
MTM: Microstate Transition Matrices
EP: Entropy Production
UWS: Unresponsive Wakefulness Syndrome
VS: Vegetative State
MCS: Minimally Conscious State
EMCS: Emergent Minimally Conscious State
GFP: Global Field Power
GEV: Global Explained Variance
GMD: Global Map Dissimilarity
HC: Healthy Controls
fMRI: Functional Magnetic Resonance Imaging
TM: Transition Matrices
LG Paradigm: Local-Global

## Footnotes

1
https://github.com/wmvanvliet/mne_microstates.
