# Supplementary material for: Dynamics of EEG Microstates Change Across the Spectrum of Disorders of Consciousness
Source: Brain Topogr. 2025 Sep 13;38(6):65. doi: 10.1007/s10548-025-01142-x (PMC12431892; doi:10.1007/s10548-025-01142-x)
Supplement: Supplementary file 1 — Supplementary Material 1 [file 10548_2025_1142_MOESM1_ESM.pdf]

Supplementary Materials

Dynamics of EEG Microstates Change Across the Spectrum of Disorders of Consciousness

Dragana Manasova 1,2, Yonatan Sanz Perl 1,3, Nicolas Marcelo Bruno 4,5, Melanie Valente 1, Benjamin Rohaut 1,6, Enzo Tagliazucchi 5,7, Lionel Naccache 1,8, Federico Raimondo 9,10,+, Jacobo D. Sitt 1+

1 - Sorbonne Université, Institut du Cerveau - Paris Brain Institute - ICM, Inserm, CNRS, Paris 75013, France

2 - Université de Paris Cité, Paris, France.

3 - Department of Information and Communication Technologies, Centre for Brain and Cognition, Computational Neuroscience Group, Universitat Pompeu Fabra, Barcelona, Spain

4 - Department of Physics (University of Buenos Aires), Buenos Aires, Argentina.

5 - National Scientific and Technical Research Council (CONICET), Buenos Aires, Argentina

6 - AP-HP, Hôpital de la Pitié Salpêtrière, Neuro ICU, DMU Neurosciences, Paris, France

7 - Latin American Brain Health Institute (BrainLat), Universidad Adolfo Ibáñez, Santiago, Chile

8 - AP-HP, Hôpital Pitié-Salpêtrière, Service de Neurophysiologie Clinique, Paris, France

9 - Institute of Neuroscience and Medicine (INM-7: Brain and Behaviour), Research Centre Jülich, Germany

10 - Institute of Systems Neuroscience, Heinrich Heine University Düsseldorf, Germany

Contact: dragana.manasova@gmail.com (ORCID 0000-0002-2756-3263), jacobo.sitt@icm-institute.org

+ these authors contributed equally to this work

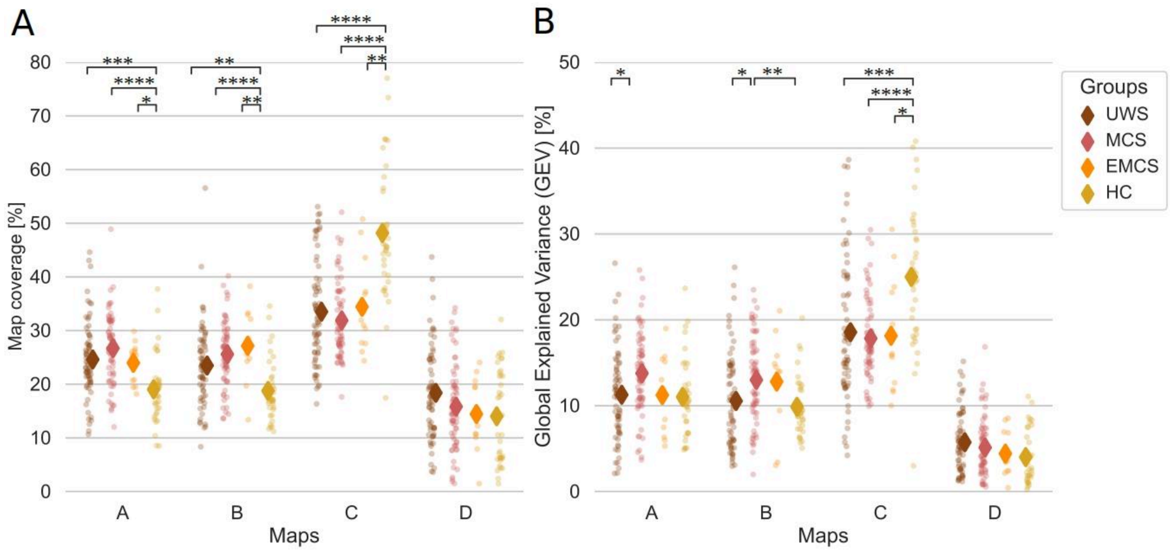

Supplementary Figure 1 Per-map distributions of the map coverage and Global Explained Variance (GEV). (A) The map coverage is shown separately for all of the different microstates (A, B, C, D). The coverage reflects the percentage of time the given microstate is dominant over the whole duration of the data. **(B)** Same as (A) but for the GEV. The statistic and the p-value of the Mann-Whitney U two-sided tests for the coverage and the GEV per map are given in Supplementary Table 1. All values are Bonferroni corrected. One dot represents one subject. The stars represent significance following Mann Whitney U tests between distributions (* p<0.05; ** p<0.01; *** p<0.001; **** p<0.0001). Abbreviations: Global Explained Variance (GEV), Unresponsive Wakefulness Syndrome (UWS), Minimally Conscious State (MCS), Emergent Minimally Conscious State (EMCS), Healthy Controls (HC).

Supplementary Table 1 Statistics and the p-value of the Mann-Whitney U two-sided tests, Bonferroni corrected, for the map coverage and the Global Explained Variance (GEV) per group per map. The values in bold pass the significance threshold of p<0.05. Abbreviations: Global Explained Variance (GEV), Unresponsive Wakefulness Syndrome (UWS), Minimally Conscious State (MCS), Emergent Minimally Conscious State (EMCS), Healthy Controls (HC).

**Map**	**Group 1**	**Group 2**	**Map coverage (statistic, p-value)**	**GEV (statistic, p-value)**
A	UWS	MCS	U(70,70)=1927, p=0.353	**U(70,70)=1735, p=0.035**
A	UWS	EMCS	U(70,14)=476, p=1	U(70,14)=483, p=1
A	UWS	HC	**U(70,37)=638, p=0.0002**	U(70,37)=1239, p=1
A	MCS	EMCS	U(70,14)=333, p=0.724	U(70,14)=348, p=1
A	MCS	HC	**U(70,37)=488, p<0.0001**	U(70,37)=830, p=0.028
A	EMCS	HC	**U(14,37)=120, p=0.0415**	U(14,37)=231, p=0.028
B	UWS	MCS	U(70,70)=1901, p=0.267	**U(70,70)=1734, p=0.034**
B	UWS	EMCS	U(70,14)=306, p=0.332	U(70,14)=361, p=1
B	UWS	HC	**U(70,37)=740, p=0.0034**	U(70,37)=1280, p=1
B	MCS	EMCS	U(70,14)=406, p=1	U(70,14)=478, p=1
B	MCS	HC	**U(70,37)=519, p<0.0001**	**U(70,37)=756, p=0.005**
B	EMCS	HC	**U(14,37)=82, p=0.0023**	U(14,37)=135, p=0.11
C	UWS	MCS	U(70,70)=2265, p=1	U(70,70)=2419, p=1
C	UWS	EMCS	U(70,14)=456, p=1	U(70,14)=488, p=1
C	UWS	HC	**U(70,37)=452, p<0.0001**	**U(70,37)=690, p=0.0009**
C	MCS	EMCS	U(70,14)=400, p=1	U(70,14)=488, p=1
C	MCS	HC	**U(70,37)=261, p<0.0001**	**U(70,37)=532, p<0.0001**
C	EMCS	HC	**U(14,37)=81, p=0.002**	**U(14,37)=121, p=0.044**
D	UWS	MCS	U(70,70)=2028, p=0.95	U(70,70)=2211, p=1
D	UWS	EMCS	U(70,14)=366, p=0.324	U(70,14)=385, p=1
D	UWS	HC	U(70,37)=957, p=0.325	U(70,37)=881, p=0.08
D	MCS	EMCS	U(70,14)=459, p=1	U(70,14)=446, p=1
D	MCS	HC	U(70,37)=1138, p=1	U(70,37)=1023, p=0.9
D	EMCS	HC	U(14,37)=242, p=1	U(14,37)=218, p=1

Supplementary Table 11 Statistics and the p-value of the Wilcoxon one-sided test against a theoretical probability of 25%, Bonferroni corrected. The values in bold pass the significance threshold of p<0.05. Abbreviations: Unresponsive Wakefulness Syndrome (UWS), Minimally Conscious State (MCS), Emergent Minimally Conscious State (EMCS), Healthy Controls (HC).

Map	Group	Map coverage Wilcoxon test against 25% (statistic, p-value)
A	UWS	W(70)=349, p=0.2
A	MCS	W(70)=406, p=0.639
A	EMCS	W(14)=17, p=1
A	HC	**W(37)=71, p=0.0006**
B	UWS	W(70)=333, p=1
B	MCS	W(70)=447, p=1
B	EMCS	W(14)=16, p=1
B	HC	**W(37)=58, p=0.0002**
C	UWS	**W(70)=102, p=0.0002**
C	MCS	**W(70)=72, p<0.0001**
C	EMCS	W(14)=0, p=0.123
C	HC	**W(37)=2, p<0.0001**
D	UWS	W(70)=132, p=0.001
D	MCS	**W(70)=82, p<0.0001**
D	EMCS	W(14)=0, p=0.123
D	HC	**W(37)=19, p<0.0001**

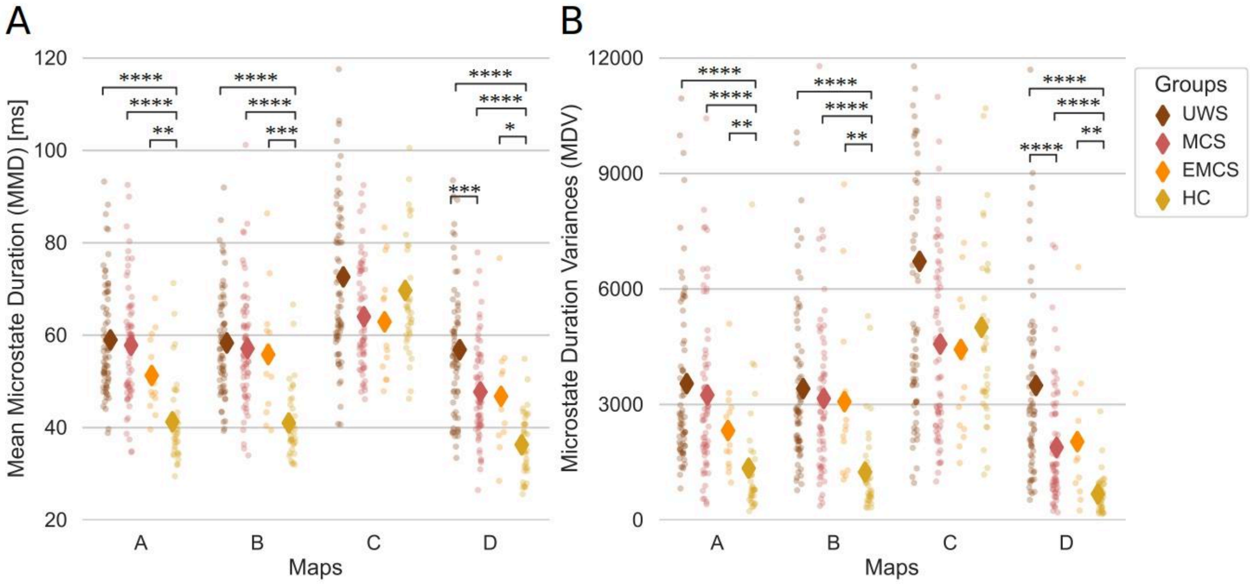

Supplementary Figure 2 Per-map distributions of the Mean Microstate Durations (MMD) and the Microstate Duration Variances (MDV). (A) The MMD are shown separately for all of the different microstates (A, B, C, D). **(B)** Same as (A) but for the MDV. The statistic and the p-value of the Mann-Whitney U two-sided tests for the MMD and the MDV per map are given in Supplementary Table 2. All values are Bonferroni corrected. One dot represents one subject. The stars represent significance following Mann Whitney U tests between distributions (* $p<0.05$; ** $p<0.01$; *** $p<0.001$; **** $p<0.0001$). Abbreviations: Mean Microstate Duration (MMD), Microstate Duration Variance (MDV), milliseconds (ms), Unresponsive Wakefulness Syndrome (UWS), Minimally Conscious State (MCS), Emergent Minimally Conscious State (EMCS), Healthy Controls (HC).

Supplementary Table III Statistics and the p-value of the Mann-Whitney U two-sided tests, Bonferroni corrected, for the Mean Microstate Durations (MMD) and the Microstate Duration Variances (MDV) per group per map. The values in bold pass the significance threshold of p<0.05. Abbreviations: Mean Microstate Duration (MMD), Microstate Duration Variance (MDV), milliseconds (ms), Unresponsive Wakefulness Syndrome (UWS), Minimally Conscious State (MCS), Emergent Minimally Conscious State (EMCS), Healthy Controls (HC).

Map	Group 1	Group 2	MMD (statistic, p-value)	MDV (statistic, p-value)
A	UWS	MCS	U(70,70)=2354, p=1	U(70,70)=2330, p=1
A	UWS	EMCS	U(70,14)=289, p=0.193	U(70,14)=333, p=0.724
A	UWS	HC	**U(70,37)=258, p<0.0001**	**U(70,37)=314, p<0.0001**
A	MCS	EMCS	U(70,14)=327, p=0.613	U(70,14)=365, p=1
A	MCS	HC	**U(70,37)=324, p<0.0001**	**U(70,37)=436, p<0.0001**
A	EMCS	HC	**U(14,37)=87, p=0.0035**	**U(14,37)=91, p=0.0049**
B	UWS	MCS	U(70,70)=2293, p=1	U(70,70)=2320, p=1
B	UWS	EMCS	U(70,14)=419, p=1	U(70,14)=413, p=1
B	UWS	HC	**U(70,37)=226, p<0.0001**	**U(70,37)=306, p<0.0001**
B	MCS	EMCS	U(70,14)=445, p=1	U(70,14)=434, p=1
B	MCS	HC	**U(70,37)=308, p<0.0001**	**U(70,37)=399, p<0.0001**
B	EMCS	HC	**U(14,37)=68, p=0.0007**	**U(14,37)=78, p=0.0017**
C	UWS	MCS	U(70,70)=1799, p=0.08	U(70,70)=1823, p=0.108
C	UWS	EMCS	U(70,14)=336, p=0.78	U(70,14)= 330, p=0.666
C	UWS	HC	U(70,37)=1214, p=1	U(70,37)=1054, p=1
C	MCS	EMCS	U(70,14)=459, p=1	U(70,14)=445, p=1
C	MCS	HC	U(70,37)=1021, p=0.88	U(70,37)=1192, p=1
C	EMCS	HC	U(14,37)=196, p=1	U(14,37)=212, p=1
D	UWS	MCS	**U(70,70)=1499, p=0.0009**	**U(70,70)=1342, p<0.0001**
D	UWS	EMCS	U(70,14)=259, p=0.068	U(70,14)=291, p=0.206
D	UWS	HC	**U(70,37)=226, p<0.0001**	**U(70,37)=140, p<0.0001**
D	MCS	EMCS	U(70,14)=456, p=1	U(70,14)=449, p=1
D	MCS	HC	**U(70,37)=457, p<0.0001**	**U(70,37)=468, p<0.0001**
D	EMCS	HC	**U(14,37)=101, p=0.011**	**U(14,37)=84, p=0.0028**

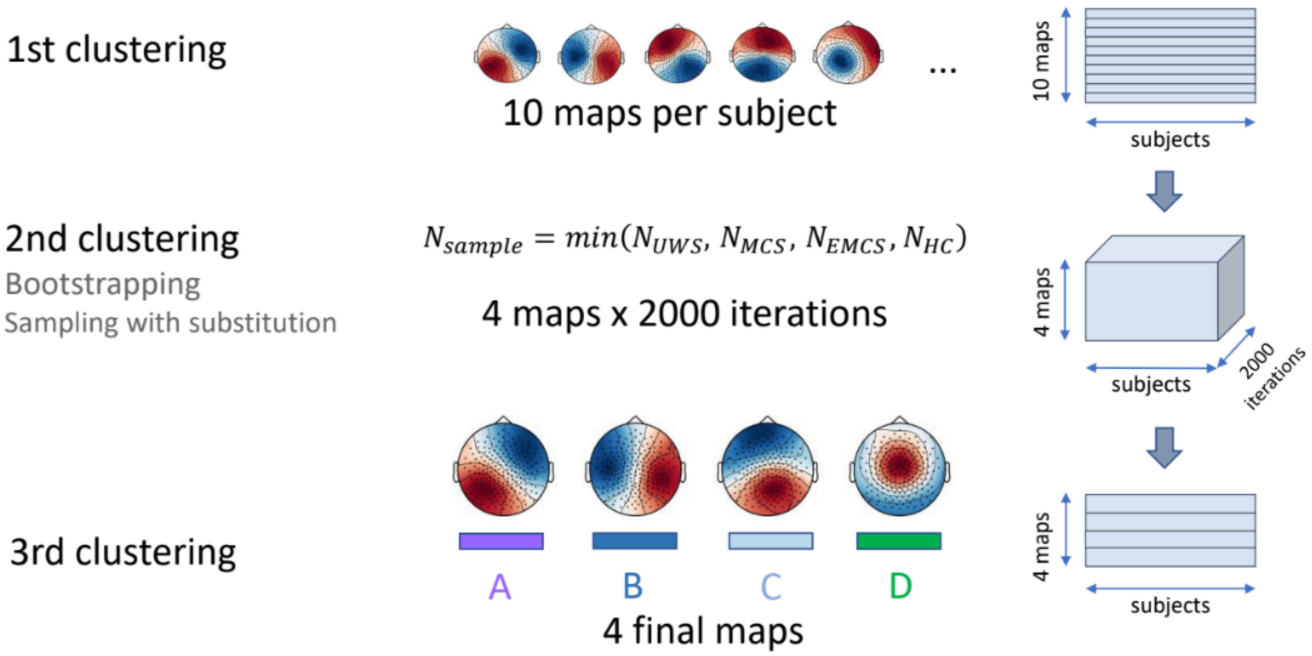

Supplementary Figure 3 Graphical representation of the 3-level clustering. In the first level, we set the number of clusters k to 10. This is done on a single subject level. The 10 resulting maps from the modified k-means clustering are fed to a second-level clustering where k=4. In this step, we do a bootstrapping due to the unbalanced sample sizes. We take samples with repetition from each group with a sample number equal to the largest sample size per group - in our case N_{EMCS}=14. We do this clustering on the sub-samples 2000 times. Thus we obtain 4 x 2000 maps, which we give to a 3rd-level clustering where k=4. These last 4 maps are back-fitted to the time series and thus the segmentation of the microstates is obtained. Abbreviations: Unresponsive Wakefulness Syndrome (UWS), Minimally Conscious State (MCS), Emergent Minimally Conscious State (EMCS), Healthy Controls (HC).
